# Correlation of systemic immune-inflammation index and moderate/major depression in patients with depressive disorders: a large sample cross-sectional study

**DOI:** 10.3389/fpsyt.2023.1159889

**Published:** 2023-05-18

**Authors:** Shu Cui, Juanjuan Li, Yun Liu, Gaofeng Yao, Yanhai Wu, Zhiwei Liu, Liang Sun, Longlong Sun, Huanzhong Liu

**Affiliations:** ^1^Department of Psychiatry, Third People’s Hospital of Fuyang, Fuyang, Anhui, China; ^2^Department of Psychiatry, Chaohu Hospital Affiliated to Anhui Medical University, Hefei, Anhui, China; ^3^School of Mental Health and Psychological Sciences, Anhui Medical University, Hefei, Anhui, China

**Keywords:** depression disorder, systemic immune-inflammation index, moderate/major depression, trait marker, risk factors

## Abstract

**Objective:**

To evaluate the clinical value of systemic immune-inflammation index (SII) based on peripheral blood neutrophil, lymphocyte, and platelet count in evaluating the subtype and severity of depression in patients with depressive disorder.

**Methods:**

This retrospective cohort study was conducted in the Third People’s Hospital of Fuyang City from January 1, 2020 to December 31, 2022. The data included sociodemographic information at admission, clinical data, discharge diagnosis and inflammatory markers. Patients were divided into low SII group and high SII group according to the optimal threshold of SII determined by receiver operating characteristic curve (ROC curve). Binary logistic regression was used to analyze the correlation between moderate/major depression and SII level.

**Results:**

Compared to the low SII group, the high SII group had a higher age level (*χ*^2^ = 7.663, *p* = 0.006), more smokers (*χ*^2^ = 9.458, *p* = 0.002), more moderate/major depression patients (*χ*^2^ = 45.645, *p* < 0.001), and a higher proportion of patients with accompanying somatic symptoms (*χ*^2^ = 14.867, *p* < 0.001). In the final logistic regression model, after controlling for confounding factors, SII at admission was significantly associated with moderate/major depression [*β* =1.285, *p* < 0.001; odds ratio (95% confidence intervals) = 3.614 (2.693–4.850)]. Patients with high SII scores were 3.614 times more likely to have moderate/severe depression than those with low SII scores. We propose a cut-off value of SII =540.78 (sensitivity = 36.4% and specificity = 80.3%) according to the maximum Youden index.

**Conclusion:**

Our research indicates that SII may be a useful, repeatable, convenient, and affordable index to identify moderate/major depression in depressive disorder.

## 1. Introduction

Depression is a frequent illness severely limiting psychosocial functioning and reducing quality of life. In 2008, the World Health Organization ranked depression as the third leading cause of disease burden worldwide and predicts that by 2031, depression will rank first (1). Depressive disorders are heterogeneous syndromes in which individuals affected by them differ significantly in their symptom profile and response to treatment, and the detection and diagnosis of depression often pose a challenge for clinicians. Recognition of this heterogeneity in depression has long led researchers to identify meaningful and valid symptom-based subtypes to guide etiologic research and to help develop more targeted interventions and treatment programs (2). Over the past decade, the use of the 10th edition of the International Classification of Diseases (ICD-10) by most general medical practices and clinicians in China to assign diagnostic categories or subtypes to individuals is clinically necessary to elucidate the heterogeneity and severity of depressive symptoms. Three different forms of depressive episodes are described in ICD-10: mild, moderate, and major. The distinction between the three relies on complex clinical judgments, including the number and type of symptoms and their severity. Approximately 30% of patients do not respond to first-line antidepressants, so there is an urgent need to explore new therapeutic targets (3).

An increasing number of studies suggest the involvement of immune-inflammatory responses as well as neuroinflammation in the pathophysiological processes of depression, while an increasing number of data on the relationship between these processes and classical neurotransmitters, the hypothalamic-pituitary-adrenal axis (HPA) and inflammatory factors have been reported (4–7). Experimental animal studies in animal models of immune inflammation and depression have found that lipopolysaccharide (LPS), interleukin-6 (IL-6), tumor necrosis factor-α (TNF-α), interferon-γ (IFN-γ) and other pro-inflammatory factors induce inflammatory responses in the body leading to a lack of pleasure as well as depression-like behavior in mice (8, 9). Paroxetine directly affects the macrophage response to inflammatory stimuli, for example, paroxetine significantly inhibits LPS-induced IL-6 production (9), which may be one of the mechanisms of action for its therapeutic effect on depression. Several clinical trials have shown widespread activation of the immune inflammatory response in groups of patients with depressive disorders (10, 11), and meta-analyses have shown that antidepressants reduce some markers of peripheral inflammation, such as significantly reduced levels of peripheral blood interleukin-1β (IL-1β), IL-6, TNF-α and interleukin-10 (IL-10) (10).

The baseline systemic immune-inflammation index (SII) is an indicator of immune response and systemic inflammation based on peripheral blood platelet, lymphocyte, and neutrophil counts, and is a simple way to objectively reflect the balance between inflammatory and immune responses (12, 13). SII calculated using the hematological findings of ischemic stroke patients at the time of admission, has shown good results in predicting post-stroke depression (PSD) at one month (14). Large-scale studies in people recovering from COVID have shown that SII is significantly and positively associated with depression and anxiety scores (15). SII measured in the emergency department, predicts more severe autonomic depression and post-traumatic stress disorder (16). However, there is a paucity of relevant studies on the potential evaluative value of SII for diagnostic subtypes in patients with depressive disorders to date.

The purpose of this study is to explore the clinicopathological characteristics of depressed patients with high SII, to explore the potential of the SII index in the assessment of diagnostic subtypes in patients with depressive disorders, and to provide a simpler diagnostic assessment tool for the clinic from a new perspective.

## 2. Materials and methods

### 2.1. Study participants

This is a retrospective cohort study. Patients who were hospitalized and diagnosed with the depressive disorder at the Third People’s Hospital of Fuyang City from January 2020 to December 2022 were consecutively enrolled. Depressive disorders were defined and diagnosed by psychiatrists according to 10^th^ revision Diagnostic Criteria for Mental Behavior Disorders of International Statistical Classification of Diseases and Related Health Problems (ICD-10). The Hamilton Depression Scale (HDRS) is used to assess the severity of depressive symptoms. Hamilton total score was evaluated as follows: 8–16: mild depression; 17–23: moderate depression; and ≥24: major depression (17). The administration of HDRS involves structured interviews with patients, and each item is rated based on patient response. Interviews are conducted by trained psychiatrists, among whom there has been consistent training. Prior to patient assessment using ICD-10 and the HDRS, psychiatrists were trained in consistency to assure consistency among psychiatrists. The inclusion criteria in this study were as follows: (1) meeting the diagnostic criteria of depressive disorder in ICD-10; (2) age ≤ 85 years old. Exclusion criteria were as follows: (1) psychiatric disorders other than depressive disorders; (2) comorbid severe physical diseases; (3) pregnant and lactating women; (4) suffering from systemic or local inflammatory diseases and inflammatory immune diseases.

This study was approved by the Ethics Committee of the Third People’s Hospital of Fuyang City (Ethics Approval No.: 2019-340-07) and was performed in accordance with the ethical standards of the Declaration of Helsinki. Patients who met the criteria had signed a written informed consent and were allowed to voluntarily withdraw from this study at any time. A total of 1,478 patients were initially enrolled in this study, of whom 102 met the exclusion criteria and 64 had insufficient baseline clinical information, and 1,312 patients were finally included in the study as study subjects.

### 2.2. Clinical data collection

Baseline demographic characteristics (gender, age, marital status, education level, smoking history), clinical manifestations (whether accompanied by somatic symptoms, psychiatric symptoms, whether this was a first episode or a relapse), discharge diagnosis, and hematological data on admission (lymphocyte count, neutrophil count, platelet count) were collected from the medical records. Blood samples were obtained from the cephalic vein of participants after fasting for at least 8 h between 6:00 and 8:00 a.m., and were collected in EDTA tubes. The complete blood counts, which included measurements of total white blood cells, neutrophils, lymphocytes, monocytes, and platelets, were analyzed using a Mindray BC-5380/BC-5180 Hematology Analyzer (Shenzhen, China). Subsequently, SII, NLR, MLR, and PLR were calculated using the appropriate formulas. SII = platelet count × neutrophil count/lymphocyte count. Neutrophil-to-lymphocyte ratio (NLR) = neutrophil count/lymphocyte count. Platelet-to-lymphocyte ratio (PLR) = platelet count/lymphocyte count. Monocyte-to-lymphocyte ratio (MLR) = monocyte count/lymphocyte count.

### 2.3. Statistical analysis

All data were statistically analyzed using SPSS 26.0 software. Data are presented as frequencies and percentages for categorical variables and as mean (standard deviation) for numeric variables. Analysis of variance (ANOVA) or chi-square test or Mann–Whitney *U* test were used to compare demographic and clinicopathological features among each group. A receiver operating characteristic (ROC) curve analysis was performed to determine the cut-off point for biomarkers to detect moderate/major depression. The optimal cut-off point is the point on the ROC curve that corresponds to the maximum Youden index. The area under the ROC curve (AUC) was performed to determine the predictive value of various indicators for moderate/major depression. A binary logistic regression model (enter) was estimated to determine the independent factors associated with moderate/major depression in patients with depressive disorder. The level of statistical significance was set at *α* = 0.05 (two-sided).

## 3. Results

### 3.1. Demographic characteristics

A total of 1,312 patients with depressive disorder were enrolled according to the inclusion and exclusion criteria. On average, the age of the entire sample was 41.69 years old (SD = 20.88; range 9 to 81), most patients were female (917 cases, 91.3%), besides, more than half of patients were unmarried (749 cases, 57%) and most had no history of smoking (1,201 cases, 92%). SII mean was 462.83 (SD = 285.40; Range 68.87 to 1886.67). Among them, 591 patients (45%) were moderate or major depression patients, and 721 patients (55%) were non-moderate or major depression patients, according to the admission diagnosis. Table 1 presents demographic and clinical characteristics by diagnostic group. There were no significant differences in gender, marital status, and smoking history between the two groups. Age, education, depression, concomitant psychotic symptoms, concomitant symptoms, SII, NLR, PLR, SII, neutrophils, leukocytes, and platelets were significantly different among the groups.

**Table 1:** Demographic and clinical characteristics of the sample.

Variables	Total sample (*n* = 1,312)	Non-moderate/major depression (*n* = 721)	Moderate/major depression (*n* = 591)	*F* or *χ*^2^ or *Z*	*p*
Age, mean (SD)	41.69 (20.88)	44.51 (20.75)	38.24 (20.52)	30.003^a^	<0.001
Gender, *n* (%)				0.013	0.910
Female	917 (69.9)	503 (69.8)	414 (70.1)		
Male	395 (30.1)	218 (30.2)	177 (29.9)		
Marital status, *n* (%)				2.682	0.101
Married	563 (42.9)	324 (44.9)	239 (40.4)		
Others	749 (57.1)	397 (55.1)	352 (59.6)		
Education, *n* (%)				5.809	0.016
Junior high school and below	565 (43.1)	332 (46.0)	233 (39.4)		
Senior high school and above	747 (56.9)	389 (54.0)	358 (60.6)		
Smoking history, *n* (%)				1.948	0.163
No	1,201 (91.5)	667 (92.5)	534 (90.4)		
Yes	111 (8.5)	54 (7.5)	57 (9.6)		
Depressive episode, *n* (%)				44.859	<0.001
First episode	1,227 (93.5)	704 (97.6)	523 (88.5)		
Relapse	85 (6.5)	17 (2.4)	68 (11.5)		
Concomitant psychotic symptoms, *n* (%)				289.461	<0.001
No	1,108 (84.5)	720 (99.9)	388 (65.7)		
Yes	204 (15.5)	1 (0.1)	203 (34.3)		
Concomitant somatic symptom, *n* (%)				9.706	0.002
No	1,231 (93.8)	690 (95.7)	541 (91.5)		
Yes	81 (6.2)	31 (4.3)	50 (8.5)		
SII, mean (SD)	462.83 (285.40)	431.26 (275.81)	501.34 (292.32)	5.331^b^	<0.001
NLR, mean (SD)	1.99 (1.12)	1.92 (1.04)	2.07 (1.21)	−2.514^b^	0.012
MLR, mean (SD)	0.16 (0.09)	0.16 (0.09)	0.16 (0.10)	1.117^a^	0.291
PLR, mean (SD)	130.31 (49.63)	127.66 (47.79)	133.54 (51.65)	5.292^a^	0.022
Neutrophils, mean (SD)	3.50 (1.43)	3.32 (1.33)	3.72 (1.52)	−5.222^b^	<0.001
Leukocytes, mean (SD)	5.88 (1.74)	5.65 (1.63)	6.16 (1.83)	−5.252^b^	<0.001
Platelets, mean (SD)	234.26 (66.69)	225.49 (63.91)	244.96 (68.49)	28.245^a^	<0.001

SII, systemic immune-inflammation index; NLR, neutrophil-to-lymphocyte ratio; PLR, platelet-to-lymphocyte ratio; MLR, monocyte-to-lymphocyte ratio.

a One-way analysis of variance (ANOVA).

b Mann–Whitney *U* test.

### 3.2. Baseline SII and optimal thresholds

**Figure 1:**
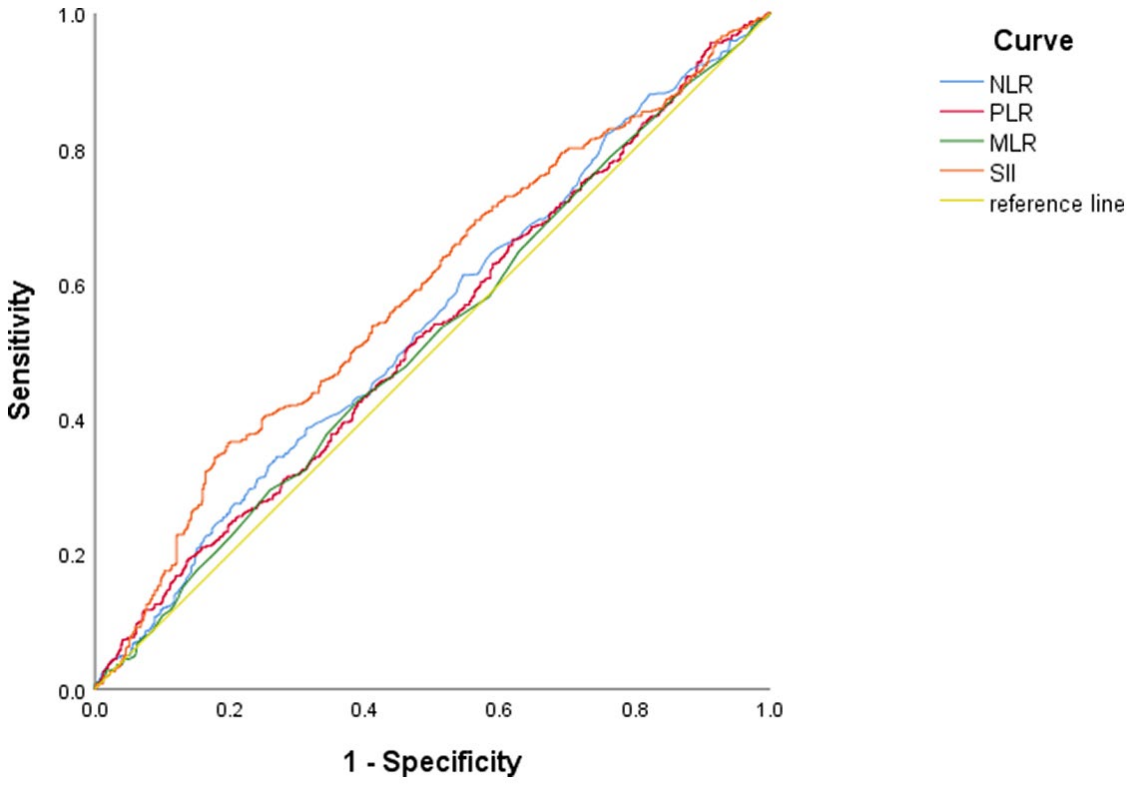
Receiver operation characteristic curve.

ROC curve (Figure 1 and Table 2) shows that the AUC value is 0.585 (95% CI: 0.554–0.616, *p* < 0.01). The optimal cut-off point is the point on the ROC curve that corresponds to the maximum Youden index. The optimal cut-off point of SII to determine moderate/major depression in patients with the depressive disorder was 540.78, the sensitivity was 0.364, the specificity was 0.803, and the Youden’s index was 0.167. Patients were grouped according to this optimal cutoff value: 357 (27%) patients in the high SII group (SII ≥ 540.78) and 955 (73%) patients in the low SII group (SII < 540.78). When NLR, MLR and PLR were used for ROC analysis, the areas under the curve were 0.54, 0.517 and 0.527, respectively (Table 2).

**Table 2:** ROC curve analysis.

Variables	Area under the curve	Standard error	*p*	95% CI
NLR	0.540	0.016	0.012	0.509–0.572
PLR	0.527	0.016	0.095	0.495–0.558
MLR	0.517	0.016	0.286	0.486–0.548
SII	0.585	0.016	<0.001	0.554–0.616

NLR, neutrophil-to-lymphocyte ratio; PLR, platelet-to-lymphocyte ratio; MLR, monocyte-to-lymphocyte ratio; SII, systemic immune-inflammation index.

### 3.3. Relationship between baseline SII and clinicopathologic features

Compared with the low SII group, the high SII group had a higher age of onset of depressive disorder (*χ*^2^ = 7.663, *p* = 0.006), more smokers (*χ*^2^ = 9.458, *p* = 0.002), more moderate/major depression (*χ*^2^ = 45.645, *p* < 0.001), and a lower number of relapse (*χ*^2^ = 9.343, p = 0.002), and the proportion of patients with somatic symptoms was higher (*χ*^2^ = 14.867, *p* < 0.001), the difference was statistically significant. The levels of neutrophils, leukocytes, platelets, SII, NLR, MLR, PLR were significantly higher in the high SII group compared to the low SII group, with statistical significance (all *p* < 0.05). There were no statistically significant differences in gender, age, education level and whether there were accompanying psychiatric symptoms between the two groups (all *p* > 0.05), as shown in Table 3.

**Table 3:** Comparison of clinicopathologic features of patients with depressive disorder between the two groups.

Clinicopathologic features	Low SII group (*n* = 955)	High SII group (*n* = 357)	*χ* ^2^	*p*
Gender, *n* (%)			0.005	0.944
Female	668 (69.9)	249 (69.7)		
Male	287 (30.1)	108 (30.3)		
Age			7.663	0.006
<40	448 (46.9)	137 (38.4)		
≥40	507 (53.1)	220 (61.6)		
Marital status			1.834	0.176
Married	399 (41.8)	164 (45.9)		
Others	556 (58.2)	193 (54.1)		
Education			0.009	0.926
Junior high school and below	412 (43.1)	153 (42.9)		
Senior high school and above	543 (56.9)	204 (57.1)		
Smoking history			9.458	0.002
No	888 (93.0)	313 (87.7)		
Yes	67 (7.0)	44 (12.3)		
Diagnostic subtype of depression			45.645	<0.001
Non-moderate/major depression	579 (60.6)	142 (39.8)		
Moderate/major depressive	376 (39.4)	215 (60.2)		
Depressive episode			9.343	0.002
First attack	881 (92.3)	346 (96.9)		
Relapse	74 (7.7)	11 (3.1)		
Concomitant psychotic symptoms			0.185	0.668
No	804 (84.2)	304 (85.2)		
Yes	151 (15.8)	53 (14.8)		
Concomitant somatic symptom			14.867	<0.001
No	911 (95.4)	320 (89.6)		
Yes	44 (4.6)	37 (10.4)		
SII, mean (SD)	325.82 (107.90)	829.32 (289.33)	−27.91^a^	<0.001
NLR	1.54 (0.55)	3.18 (1.36)	−24.18^a^	<0.001
MLR	0.14 (0.08)	0.21 (0.11)	−12.36^a^	<0.001
PLR	113.20 (34.40)	176.09 (54.89)	−19.59^a^	<0.001
Neutrophils	2.97 (0.91)	4.92 (1.59)	−21.02^a^	<0.001
Leukocytes	5.43 (1.39)	7.07 (2.01)	−14.10^a^	<0.001
Platelets	219.05 (59.05)	274.93 (68.98)	−13.15^a^	<0.001

Low SII group SII < 540.78; high SII group SII ≥ 540.78; SII, systemic immune-inflammation index; NLR, neutrophil-to-lymphocyte ratio; PLR, platelet-to-lymphocyte ratio; MLR, monocyte-to-lymphocyte ratio.

a Mann–Whitney *U* test.

### 3.4. Binary logistic regression analysis of influencing factors of moderate/major depression subtype in patients with depressive disorder

Moderate/major depression were used as the dependent variable (moderate/major depressive was 1, non-moderate/major depressive was 0), marital status (married was 1, other was 0), age classification (≥40 years old: 1, < 40 years old: 0), gender (male was 1, female was 0), education level (high school education or above was 1, Junior high school or below education: 0), smoking (smoking history: 1; no smoking history: 0), somatic symptoms (positive: 1; negative: 0), psychotic symptoms (positive: 1; negative: 0), recurrent depressive disorder (recurrent: 1; first episode: 0), SII (SII ≥ 540.78: 1; SII < 540.78: 0) as independent variable, binary multi-factor logistic regression analysis (Table 3) was performed, and the results showed that concomitant somatic symptoms (*p* < 0.001, OR = 4.09), concomitant psychotic symptoms (*p* < 0.001, OR = 574.365), relapse (*p* < 0.001, OR = 14.312), age (*p* = 0.022, OR = 0.692), SII level (*p* < 0.001, OR = 3.614) was significantly associated with moderate/major depression. SII ≥ 540.78 was a risk factor for moderate/major depression in patients with depressive disorder (p < 0.001), as shown in Table 4.

**Table 4:** Binary logistic regression analysis of influencing factors of moderate/major depression subtypes in patients with depressive disorder.

	*B*	Wald statistic	*p*	OR	95% CI of OR
Marriage statues	−0.212	1.963	0.161	0.809	0.602–1.088
Age	−0.368	5.267	0.022	0.692	0.505–0.948
Gender	−0.006	0.001	0.970	0.994	0.714–1.383
Education	0.019	0.017	0.897	1.019	0.763–1.362
Smoking statues	−0.367	1.573	0.210	0.693	0.390–1.230
Concomitant somatic symptoms	1.409	28.622	<0.001	4.090	2.441–6.853
Concomitant psychotic symptoms	6.353	39.764	<0.001	574.365	79.725–4137.922
Relapse	2.661	83.585	<0.001	14.312	8.090–25.320
SII	1.285	73.293	<0.001	3.614	2.693–4.850

Low SII group SII < 540.78; high SII group SII ≥ 540.78; SII is the index of systemic immune inflammation.

## 4. Discussion

Depressive disorder is a disease with high morbidity and disability rate. This study showed that patients with depressive disorder in the high SII group were older, had a higher odds of a history of smoking history, had a higher proportion of moderate/major depression, and had a higher proportion of accompanying somatic symptoms. At the same time, high SII is a risk factor for moderate/major depression in patients with depressive disorder.

This study demonstrated the potential application value of SII index on admission to determine the severity of depression. The SII index objectively reflects the balance between the inflammatory and immune responses of the body. It provides a simple, rapid, and inexpensive way to measure the level of inflammation. The SII index can be calculated by the simple parameters in the blood routine upon admission. For patients with high SII depression, the occurrence of moderate/major depression can be warned at an early stage. The incidence of suicide, somatic, and psychotic symptoms in patients with moderate/major depression are relatively high. Therefore, individualized treatment programs including psychological and physical therapy can be actively adopted in the follow-up treatment to reduce the incidence of suicide and improve the clinical cure rate (1). On the other hand, currently used antidepressants target and promote the neurotransmitter effects of monoamine, yet approximately 30% of patients do not respond to these drugs, so there is an urgent need to explore new therapeutic targets (3). Some clinical studies have reported increased levels of inflammatory cytokines and neutrophils in the blood of patients with major depression, and higher levels of interleukin 6 in the blood of drug-resistant patients (18, 19). A meta-analysis of whole blood gene expression in patients with major depression revealed upregulation of neutrophil-related genes (20). These findings may support the role of neuroinflammation in the etiology of major depression or treatment-resistant depression, and targeting this pathway may have value in the treatment of depression.

The close relationship between SII and depression has been verified in many patients with other physical diseases complicated with depression, which is consistent with the results of this study, indicating that SII has potential application value in the assessment of depression or anxiety related to physical diseases. For example, among patients with ischemic stroke, SII, platelet/lymphocyte ratio (PLR), and neutrophil/lymphocyte ratio (NLR) increased at admission, in particular, increased SII was significantly associated with the occurrence of post-ischemic stroke depression one month later, which may provide some prognostic clues for early detection of post-ischemic stroke depression (14). In the tuberculosis population, patients with symptoms of depression or anxiety had poorer cellular immune status and stronger inflammatory response than patients without symptoms of anxiety or depression, and a higher SII was significantly associated with symptoms of depression or anxiety (*p* < 0.05) (21). A study of 2,566 patients with diabetes (including 370 patients diagnosed with comorbidity depression) showed that high SII levels were an independent risk factor for diabetic depression (OR = 1.347, 95%CI: 1.031–1.760, *p* = 0.02). The researchers further validated the relationship between SII and diabetic depression by using propensity matching analysis, and SII may be an accessible and cost-effective strategy for identifying depression in diabetic patients (22). Data from a follow-up study of persistent psychopathology and cognitive impairment in COVID-19 survivors showed that baseline SII predicted self-rated depressive symptoms and cognitive impairment at three-month follow-up, and changes in SII predicted changes in depression severity during follow-up. Baseline SII can predict neurocognitive impairments associated with the severity of depressive psychopathology, including processing speed, verbal memory and fluency, and psychomotor coordination. The investigators hypothesize that COVID-19 may lead to chronic systemic inflammation, redisplaying patients to persistent depression and related neurocognitive dysfunction (23).

More and more animal models and clinical studies of depression suggest that immunoinflammatory and neuroinflammatory pathways are involved in the pathophysiological processes of depression. Studies on immune inflammation and depression animal models have shown that systemic inflammatory response induced by lipopolysaccharide, tumor necrosis factor α and other pro-inflammatory factors can lead to depression-like behavior in mice (24–26). Compared with healthy controls, the levels of interleukin-4, interleukin-6, and interleukin-10 changed significantly in major depressive disorder patients (27). The role of inflammation in the etiology and exacerbation of depression is further supported by studies showing that increased levels of interleukin-6 in childhood increase the risk of depression later in life (26), while postmortem examination of the brains of depressed patients shows significant neuroinflammation and widespread activation of microglia (28).

Platelets are a specific inflammatory indicator. Platelets are increasingly recognized to play an important role in inflammation and as a putative bridge between mental illness and inflammatory response. Platelet overactivation has been observed in patients with depressive disorders, and platelet parameters have potential predictive power for major depression and bipolar disorder (29). Inflammatory mediators generate activated platelets by activating macrophages. On the one hand, they activate dense particles inside platelets to release serotonin; on the other hand, activating platelets further enhances vascular permeability by releasing pro-inflammatory factors, gathering monocytes, and enhancing inflammatory response (30). Serotonin and pro-inflammatory factors derived from platelet activation are involved in initiating, maintaining, and regulating inflammatory responses, which play an important role in the occurrence and development of depression (30). Thus, platelet activation is involved in systemic inflammatory responses and pathophysiological processes of depression.

Peripheral blood neutrophil/lymphocyte ratio (NLR) is a component of the SII formula and a simple measure to evaluate the inflammatory status and can economically and easily detect the activation of inflammatory systems. More and more studies have shown that the level of NLR in patients with depressive disorder is higher than that in healthy control volunteers, and is closely related to the degree of depression. For example, one study showed significantly higher levels of NLR in patients with affective disorders, and may assess the course of affective disorders (31). Another study also revealed that non-medicated patients with major depressive episodes had higher levels of NLR than healthy controls (32). In male patients with depression, NLR was significantly correlated with depressive symptoms, and the higher the NLR, the more severe the symptoms (33). NLR has become a potential biomarker of inflammation in depressed patients with suicide attempts (34), HAMD scores in depressed patients were positively correlated with NLR levels in depressed patients, with NLR ≥ 1.57 being an independent predictor of severe or very severe depression (35). In the group of adolescents with major depressive disorder, disease severity was positively correlated with NLR, which may support the hypothesis that inflammation plays an important role in the etiology of major depressive disorder in adolescents (36).

The novelty of this study lies in the identification of SII index as a potential predictor for moderate/severe depression. This suggests that monitoring SII levels could help in identifying individuals who are at risk for developing moderate/severe depression, and potentially enable early intervention and treatment. This finding also adds to the growing body of literature on the complex relationship between inflammation and mental health, and highlights the potential role of SII as a novel biomarker for depression risk assessment.

This study has the following deficiencies: (1) there is a lack of information on potential confounders, such as whether the patient was taking any psychiatric medications at the time of admission, especially antidepressants, including the dose and duration of treatment, as previously stated in the literature, antidepressants have anti-inflammatory effects (37, 38). (2) This study was a non-multicenter study; (3) the ability to detect biomarkers reflecting inflammatory and immune responses was insufficient, which could not fully reflect the pathophysiological process of depression; (4) important psychosocial stress situations related to depressive events and pre-admission treatment were not collected, such as negative life events, antidepressant medication or psychotherapy at baseline, etc. These factors may affect the diagnostic subtypes of depressive disorder. (5) SII is only calculated by the blood routine results of patients upon admission, but this indicator has dynamic changes in the course and development of the depressive disorder. In future work, we need to conduct further research on the relationship between the changes of SII at different time points and the occurrence and development of the depressive disorder.

Although this study has some limitations, our study confirms that high SII on admission is an independent risk factor for symptoms reaching moderate/major depression in patients with depressive disorder. In future clinical work, for depressed patients with high SII value upon admission, emphasis should be placed on early depression assessment, early psychological intervention and prevention, which will have a good impact on the prognosis of patients. On the other hand, rapid identification of people with high SII may lead to improvements in subsequent treatments, such as combining conventional treatments with treatments that reduce inflammation levels to treat major depression.

## Data availability statement

The raw data supporting the conclusions of this article will be made available by the authors, without undue reservation.

## Ethics statement

The studies involving human participants were reviewed and approved by Ethics Committee of the Third People’s Hospital of Fuyang City (Ethics Approval No.: 2019-340-07). Written informed consent to participate in this study was provided by the participants’ legal guardian/next of kin.

## Author contributions

SC, HL, and YL: conception and design. SC, YL, and GY: administrative support. SC, JL, YW, ZL, LiS, and LoS: collection and assembly of data. SC, GY, JL, and HL: data analysis and interpretation. SC, JL, YL, GY, YW, ZL, LiS, LoS, and HL: manuscript writing. All authors contributed to the article and approved the submitted version.

## Funding

This study was funded by the Fuyang Municipal Health Commission Research Project (FY2020xg14) and Clinical Medical Key Specialty Construction Project of Anhui Province of China [(2021)273].

## Conflict of interest

The authors declare that the research was conducted in the absence of any commercial or financial relationships that could be construed as a potential conflict of interest.

## Publisher’s note

All claims expressed in this article are solely those of the authors and do not necessarily represent those of their affiliated organizations, or those of the publisher, the editors and the reviewers. Any product that may be evaluated in this article, or claim that may be made by its manufacturer, is not guaranteed or endorsed by the publisher.
